# Macon Medical Society

**Published:** 1885-04

**Authors:** 


### MACON MEDICAL SOCIETY.

Reported for the ATLANTA MEDICAL AND SURGICAL JOURNAL.

STATED MEETING, January 20, 1885.

The President in the chair.

Essay of the evening, on Catarrhal Fever, read by Dr. Moore.

Subject for discussion:

### ORCHITIS AND EPIDIDYMITIS.

Dr. Moore—Orchitis is rare, and is usually dependent on mumps, cold or injury. Epididymitis is dependent on gonorrhœa or urethritis. Whether it is a metastasis or an extension of the inflammation is a question. Dr. Hammond uses decided doses of tartar emetic, and when the urethritis stops he starts it again with injections. I do not advise starting up the discharge again. My treatment is to wrap the testicle in a tobacco poultice and open the bowels with a saline. The cases usually last from three to seven days.

Dr. Fitzgerald—I do not believe that epididymitis is always caused by urethritis. It may come from an enlarged prostate or irritated neck of the bladder, and from injections. Much harm is done by injections. By throwing them against the cilliæ of the epithelium of the urethra they act as irritants, causing both epididymitis and orchitis. The medication should be introduced into the bladder with a catheter and allowed to run out. Suspension and rest for the testicle is the treatment.

Dr. Hall—Do your cases get well as quick as Dr. Moore's?

Dr. Fitzgerald—They don't give much more trouble. I have little confidence in medication. Nature will cure them if the cause is removed.

Dr. McHatton—I regard orchitis as a rare disease, while epididymitis is the most common disease of the testicle. Orchitis is more than apt to end very seriously, namely, by atrophy of the organ from pressure. Absolute rest with elevation of the testicle should be enforced, and if the symptoms fail to abate, resort should be had to subcutaneous section of the tunica albuginea to relieve pressure. I agree with Dr. Moore in his treatment of epididymitis. An important feature of this disease is that described by Gosselin, namely, the tubes being blocked up by inflammatory products, the patient is sometimes rendered permanently sterile.

Dr. Hall—My treatment is warm applications and belladonna externally, with quinia and morphia internally. By beginning treatment early the disease may be aborted. I recall a case of double epididymitis, who was married six years afterwards and has four children; also two other cases that have children.

Dr. Stevens—I had a case of double orchitis and double epididymitis following mumps. He was nine weeks in bed; his wife has had a child since.

Dr. McHatton—Sterility is by no means the rule, but is common enough for us to bear it in mind.

